# Cathepsin K^+^ Non-Osteoclast Cells in the Skeletal System: Function, Models, Identity, and Therapeutic Implications

**DOI:** 10.3389/fcell.2022.818462

**Published:** 2022-07-13

**Authors:** Nanyu Zou, Ran Liu, Changjun Li

**Affiliations:** ^1^ Department of Endocrinology, Endocrinology Research Center, The Xiangya Hospital of Central South University, Changsha, China; ^2^ National Clinical Research Center for Geriatric Disorders (Xiangya Hospital), Changsha, China; ^3^ Key Laboratory of Organ Injury, Aging and Regenerative Medicine of Hunan Province, Changsha, China

**Keywords:** cathepsin K, Ctsk+ cells, animal model, genetic marker, bone metabolic disease

## Abstract

Cathepsin K (Ctsk) is a cysteine protease of the papain superfamily initially identified in differentiated osteoclasts; it plays a critical role in degrading the bone matrix. However, subsequent *in vivo* and *in vitro* studies based on animal models elucidate novel subpopulations of Ctsk-expressing cells, which display markers and properties of mesenchymal stem/progenitor cells. This review introduces the function, identity, and role of Ctsk^+^ cells and their therapeutic implications in related preclinical osseous disorder models. It also summarizes the available *in vivo* models for studying Ctsk^+^ cells and their progeny. Further investigations of detailed properties and mechanisms of Ctsk^+^ cells in transgenic models are required to guide potential therapeutic targets in multiple diseases in the future.

## Introduction

As one of the most efficacious lysosomal cysteine proteases of the papain superfamily initially identified in differentiated osteoclasts (Drake et al., 1996), cathepsin K (Ctsk) is now known to be expressed in various tissues and stem/progenitor cells, such as periosteal stem cells (PSCs) (Debnath et al., 2018; Han et al., 2019), Ctsk^+^ chondroid progenitors (CCPs) (Yang et al., 2013), tendon-derived progenitor cells (TDPCs) (Feng et al., 2020), breast cancer cells, prostate cancer cells (Littlewood-Evans et al., 1997; Brubaker et al., 2003), neurons (Bernstein et al., 2007), failing heart, atherosclerotic lesions (Sukhova et al., 1998; Shi et al., 1999; Lutgens et al., 2006), bronchial and alveolar epithelial cells, alveolar macrophages (Bühling et al., 1999; Haeckel et al., 1999; Bühling et al., 2001), and white adipose tissue (Soukas et al., 2000; Chiellini et al., 2003). In addition, Ctsk plays a critical role in degrading the bone matrix and contributes to osteoclast-mediated bone resorption. Osteoporosis occurs when the Ctsk-induced bone resorption exceeds bone formation. Furthermore, Ctsk is involved in the pathogenesis of pulmonary fibrosis (Bühling et al., 2004), atherosclerotic plaque progression (Guo et al., 2009), and some tumor metastasis, such as epithelial ovarian cancer, melanoma, prostate cancer, and breast cancer (Brubaker et al., 2003; Munari et al., 2017; Petricevic et al., 2017; Fan et al., 2018; Gu et al., 2019). In this review, we mainly discuss the available animal models for studying Ctsk^+^ cells and their progeny, identity, role of Ctsk-labeled nonosteoclast cells in the skeletal system, and translational applications of Ctsk inhibitors in bone metabolic diseases.

## Cathepsin K: Collagenolytic Cysteine Protease

Ctsk is a cysteine protease of the papain superfamily, and it is mainly known for its significant resorptive function in bones (Inaoka et al., 1995; Drake et al., 1996). Osteoclasts cling to the bone surface via a specialized cell-matrix adhesion structure, the sealing zone (Takito et al., 2018). Ctsk is predominantly expressed in mature osteoclasts and is secreted along with protons into the resorption lacunae. The low pH environment of the resorption lacunae acidifies the externally mineralized component of the bone so that Ctsk exerts its proteolytic function on the exposed organic matrix. The bone extracellular organic matrix is composed of collagen Ⅰ (90%), which is formed by a triple helix with a telopeptide end (Gistelinck et al., 2016). In addition, Ctsk has a stronger collagenolytic ability than other proteases, such as matrix metalloproteinases and neutrophil serine elastase. It cleaves the triple helix across all three chains and attacks the telopeptides, while other proteases can only degrade telopeptides (Garnero et al., 1998; Kafienah et al., 1998). Once collagen Ⅰ is degraded, the products of telopeptide crosslinks from the N- and C-terminal ends are released into the circulatory system. They are detected in the blood and urine samples as markers of bone resorptive activity in osteoporosis patients. Impressively, a cross-sectional study with 1752 postmenopausal Chinese women indicated no evident connection between serum Ctsk and bone mineral density (BMD) (Gao et al., 2020). In addition, some studies have shown that Ctsk has a productive capacity for dissolving collagen Ⅱ, the primary protein marker of cartilage (Kafienah et al., 1998; Hou et al., 2001; Mort et al., 2016). The function of Ctsk in osteoclasts has been studied extensively, but its exact role in the newly discovered stem/progenitor cells remains unclear.

## *In Vivo* Models to Study Ctsk^+^ Cells and Their Progeny

The generation of transgenic models has contributed to visualizing and isolating Ctsk^+^ cells, and we can follow the action of Ctsk^+^ cells *in vivo* through reporter genes. The Cre recombinase technology can be used to genetically control the expression of additional genes under the Ctsk promoter. The interest focused initially on osteoclasts in bone remodeling, and it recently extended to other subtypes of stem/progenitor cells whose properties are consistent with the mesenchymal lineage. Although *in vivo* models remarkably target particular Ctsk^+^ cell subsets, they do overlap to a certain extent. Hence, a combination of models is preferable to study the Ctsk^+^ cells and their progeny.

### *Cathepsin K* Knockout Mice

Genomic structure and intron sizes of Ctsk in mice indicate that the initiation codon ATG is located at exon 2, and the termination codon TGA is at exon 8, which is highly similar to the human homolog. The first *cathepsin K* knockout (*Ctsk* ^*−/−*^) mouse model was established to explore the role of Ctsk in bone resorption. They inserted the neomycin phosphotransferase gene (*Neo*) cassette into a HindIII restriction site at exon 7, which mimicked a premature translational termination codon, to disrupt *Ctsk* transcription in embryonic stem cells (Gelb et al., 1996; Saftig et al., 1998). However, some abnormalities in pycnodysostosis, such as short stature and craniofacial defects, are not seen in *Ctsk* ^*−/−*^ mice. *cathepsin K* deficient mice proved to be a survivor with intact fertility. In addition, *Ctsk* ^*−/−*^ mice displayed no noticeable phenotypic abnormalities until they were 10 months old (Li et al., 2006). Another *Ctsk* ^*−/−*^ mouse model was set up by targeting the coding region of cathepsin exon 3 via homologous recombination (Gowen et al., 1999); the histological and radiographic analyses revealed osteopetrosis of the long bones and vertebrae. The *Ctsk* deletion in mice increased the RANKL/OPG ratio and mediated osteoclastogenesis (Kiviranta et al., 2005). Splenomegaly emerged in both pycnodysostosis and *Ctsk* ^*−/−*^ mouse models, thereby compensating for the reduced bone marrow space. Gowen et al. (Gowen et al., 1999) did not find any apparent abnormalities in other organs/tissues. Taken together, these mice may represent a valuable animal model for pycnodysostosis. In addition to osteoporosis, the *Ctsk* ^*−/−*^ mice are utilized to study the role of Ctsk in various skeletal disease models. In *Ctsk* ^*−/−*^ mice, a closed stabilized femoral fracture “Einhorn” model was developed to evaluate the effects of Ctsk inhibition on fracture healing (Gentile et al., 2014). Surprisingly, the *Ctsk* ^*−/−*^ mice had increased callus mineralization and improved callus strength compared to the wild-type mice (same sex and age). Kozawa et al. (Kozawa et al., 2012) reported that the progression of joint instability-induced osteoarthritis in *Ctsk* ^*−/−*^ mice was slower than that in the wild-type control group. In rheumatoid arthritis models, the C57BL/6J background *Ctsk* ^*−/−*^ mice hybridizing with human TNF-transgenic mice highlighted the crucial role of Ctsk in reducing inflammation and bone erosion (Hao et al., 2015). The bone volume of *Ctsk* ^*−/−*^-prednisolone mice indicated that Ctsk deletion in part helps rescue glucocorticoid-induced osteoporosis (GIO) (Yang et al., 2018), thereby making the Ctsk inhibitors effective in GIO prevention. Moreover, no animal models of *Ctsk* overexpression have been established to date. *Ctsk* overexpression may enhance bone resorption of osteoclasts and cause an osteoporosis phenotype, thereby further confirming the therapeutic effects of Ctsk deletion or Ctsk inhibitor on various diseases.

### *Ctsk-GFP*/*Ctsk-YFP*

To et al. (To et al., 2012) established an osteoclast reporter line in medaka wherein the *Ctsk* promoter controlled the expression of green fluorescent protein (GFP). A 3.18-kb upstream regulatory region of the medaka *Ctsk* gene, including 80 nucleotides of exon 1, was cloned in front of *mEGFP* in an I-SceI meganuclease vector. Chatani et al. used a 3-kb upstream sequence of the *Ctsk* gene, a slightly shorter promoter, to generate a *Ctsk-GFP* line comparable to the former (Chatani et al., 2011). Interestingly, *Ctsk* is endogenously expressed in various tissues, such as the heart, a single neuron in the brain, and the pineal gland, but it is not detected in transgenics. It shows selectivity in cell subsets, and the used promoter lacks regulatory elements that can drive the expression in these nonskeletal tissues. However, for the most part, *in situ* ribonucleic acid (RNA) hybridization revealed that *Ctsk:mEGFP* expression was comparable to endogenous *Ctsk* expression of the protein. The *Ctsk:mEGFP* expression in tartrate-resistant acid phosphatase-positive (TRAP^+^) cells started 12 days postfertilization (To et al., 2012). The author also used *Ctsk:mEGFP* and mCherry under the control of the *Osterix* promoter to observe the interaction between osteoclasts and osteoblasts *in vivo*. However, a medaka osteoclast reporter line that expressed *mEGFP* under the control of the *Ctsk* promoter reported revealed destructive osteoclast activities and vertebral deformations in adult medaka (To et al., 2015). After the termination of the *Ctsk* expression of the protein, fluorescence could still be detected due to the turnover of GFP. Reporter genes with an upstream floxed STOP cassette in mice help trace the lineage generations with Ctsk^+^ progenitor populations, thereby unavoidably making it difficult to distinguish the targeted Ctsk^+^ cells from the lineage-traced ones.

### *Ctsk-Cre*/*Ctsk-Cre* ^*ERT2*^

The Cre-loxP-mediated recombination system (the “Cre-loxP system”) is a powerful and broadly used genetic experimental tool for selectively deleting, exchanging, or inserting genes of interest in specific discrete cells. The “*floxed*” loci (flanking DNA segments) are readily formed in mice by targeting embryonic stem cells, although blastocysts are also workable in mice (Sauer, 1998; McLellan et al., 2017). Several *Ctsk-Cre* lines are generated to direct recombination to osteoclasts, chondrocytes, PSCs, CPCs, and TDPCs. Specifically, once phenotypic differences occur after conditionally deleting the targeted gene in Ctsk-Cre cells in the bone, identifying the exact Ctsk^+^ cell type that made the difference is controversial. Although the *Ctsk* gene promoter helps narrow the range of cells that express Cre, the potential off-target Cre expression is detected in other tissues, including the brain, colon, heart, kidneys, liver, lungs, spleen, stomach, and ovary (Chiu et al., 2004). There is no perfect solution at the moment, but the off-target effects need to be considered and rigorous tests need to be performed after a mouse model is constructed.

The *Ctsk-Cre* ^*ERT2*^ mouse model is a sophisticated genetic model based on the Ctsk-Cre model that controls *Ctsk* lineage temporally. The *Ctsk* promoter drives the expressions of Cre and estrogen receptor ligand-binding domain fusion proteins (Cre-ER-LBD). In the absence of tamoxifen, the Cre-ER-LBD fusion proteins are sequestered in the cytoplasm using heat shock protein 90 (HSP90). Tamoxifen, after injection, replaces HSP90 and binds with cre-ER fusion proteins to initiate recombination. In this process, the cre-ER is not responsive to estrogen and is not influenced by estrogen fluctuation (McLellan et al., 2017). *Cre-ER* ^*T2*^ is the most common tamoxifen-inducible *Cre* transgene, which showed ten-fold greater sensitivity to tamoxifen than *cre-ER* ^*T*^. Polymerase chain reaction, histological analyses, and E17.5 embryos showed that *Ctsk-cre-ER* ^*T2*^ was expressed predominantly in bones (Sanchez-Fernandez et al., 2012). Furthermore, this improved genetic tool avoided some embryonic and postnatal gene lethality and allowed the compilation of genes at any time during development or adulthood. However, tamoxifen served as an active, selective estrogen receptor modulator that affects bone homeostasis. It was required in the control group with the same amount to nullify the tamoxifen effect to obtain comparable consequences.

## Identity of Ctsk^+^ Cells in the Skeletal System

The abovementioned transgenic models have helped identify different subsets of Ctsk^+^ cells and their progeny. This section discusses the novel nonosteoclast Ctsk^+^ cell subsets characterized to date in the skeletal system.

### Ctsk^+^ Chondrocytes and Fibroblast-Like Synoviocytes

**FIGURE 1:**
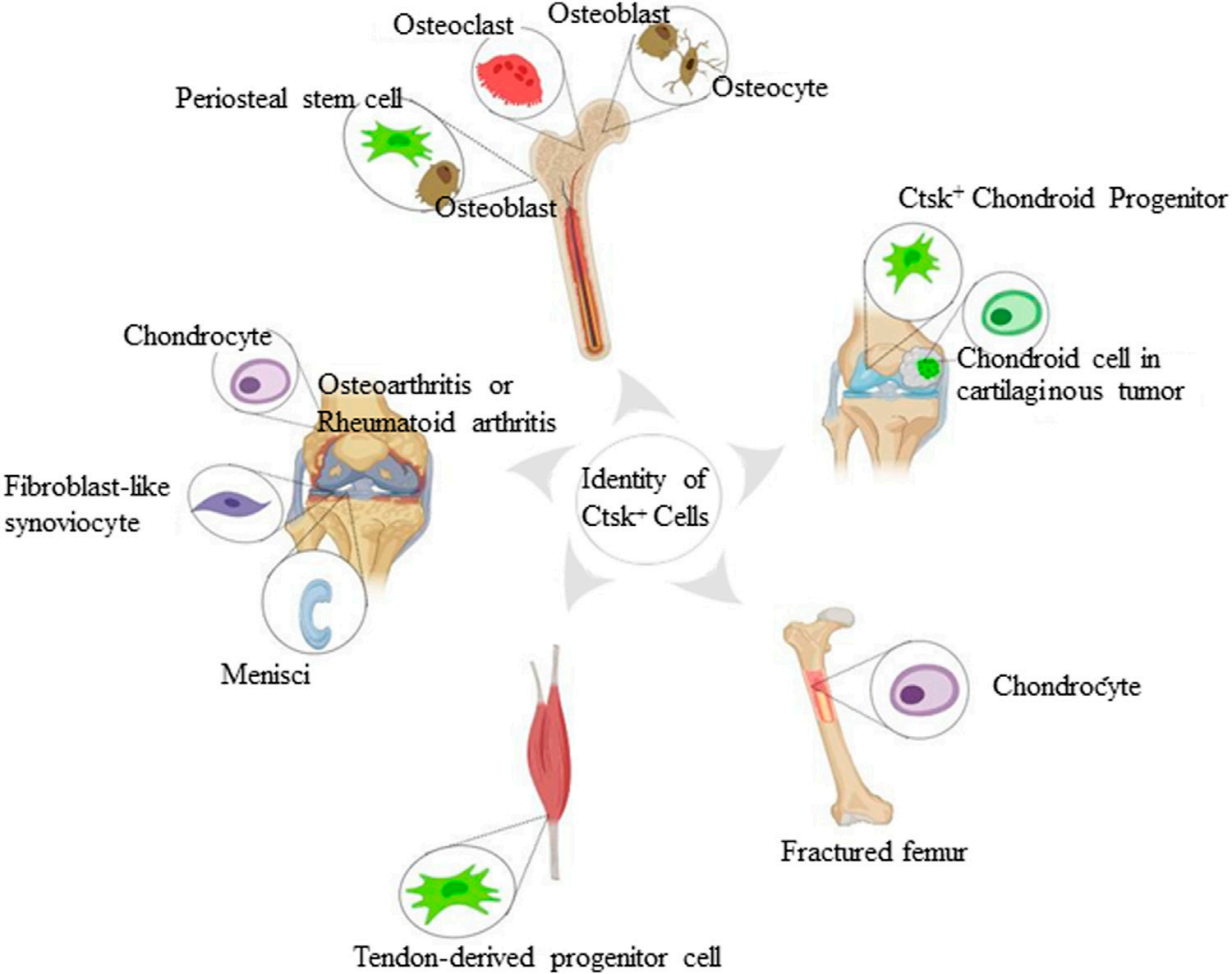
Identity of Ctsk^+^ cells found in the skeletal system. The schematic depiction shows subsets of Ctsk^+^ cells, including mature osteoclasts, osteoblasts, osteocytes, chondrocytes, and some mesenchymal stem/progenitor cells. Mature osteoclasts, osteoblasts, and osteocytes are displayed according to Ctsk immunostaining. Ctsk^+^YFP^+^ stem/progenitor cells are represented in green.

Random sequencing of expressed sequence tags from cDNA libraries and Ctsk immunostaining indicated that Ctsk is predominantly stained in mature osteoclasts. Moreover, Ctsk is granularly located at a single pole of the osteoclasts (Drake et al., 1996; Hussein et al., 2017; Debnath et al., 2018). Using the same methodology, Ctsk is moderately expressed in osteoblasts and in some osteocytes underlying the matrix of metaphyseal trabecular femoral heads. However, the *Ctsk* expression of the protein was reduced during bone matrix formation (Mandelin et al., 2006; Hussein et al., 2017; Lotinun et al., 2019). Immunohistochemical analysis conducted in the degradation area, weight-bearing areas of knee cartilage, and menisci in osteoarthritis patients showed that chondrocytes and fibroblast-like synoviocytes expressed Ctsk. Quantitative morphometric analysis showed enhanced expression compared with healthy subjects (Morko et al., 2004; Kozawa et al., 2016). However, the lack of genetic markers to identify stem/progenitor cells, such as PSCs, and the absence of distinction among various progenitor cell types restrict the further exploration of the properties of these cells (Figure 1).

### Ctsk^+^ Chondroid Progenitors

Yang et al., for the first time, found Ctsk-positive cells in extramedullary bones and confirmed the nonosteoclast nature of these cells using Ctsk-Cre transgenic mice (Yang et al., 2013). To trace and characterize this subpopulation of Ctsk^+^ cells, they used Rosa26LSL-lacZ (*R26LSL-lacZ*) or Rosa26LSL-YFP (*R26LSL-YFP*) Cre reporter mice. *Ctsk*-positive cells were a subpopulation of perichondrial cells in the “groove of Ranvier.” The perichondrial groove of Ranvier has been reported to serve as a potential reservoir of cartilaginous precursor stem cells. These cells could migrate to the growth plate and knee joint to maintain cartilage homeostasis (Fenichel et al., 2006; Karlsson et al., 2009). Furthermore, the Ctsk-yellow fluorescent protein (YFP^+^) was detected in the articular cartilage and growth plate (Yang et al., 2013). Usami et al. revealed that ZsG^+^ cells occupied the majority of cells in the perichondrial groove of Ranvier at the postnatal age of 7 days (P7) of the *Ctsk-cre;Rosa26ZsGreen* mouse system and showed up in the epiphyseal cartilage around P14. Unlike what Yang et al. had observed, these ZsG^+^ cells did not migrate to the growth plate at P28 or P70 (Usami et al., 2019). Few red fluorescent protein-positive (RFP^+^) cells in *Col2-Cre* ^*ERT*^ *;RFP* mice were detected in the perichondrial groove of Ranvier 3 or 7 days after injection, further indicating that chondrocytes in the growth plate did not originate from the perichondrial groove of Ranvier from the neonatal to the growth stage. These inconsistent arguments may be attributed to different reporter mice, and cells in the perichondrial groove of Ranvier are not the significant sources that produce cells in the growth plate during postnatal growth under physiological conditions. However, they may increase the odds of differentiating into chondrocytes and provide osteoblasts under pathological circumstances. Interestingly, CCPs have the capacity of *in vitro* osteogenesis, adipogenesis, and chondrogenesis*.* They display CD44, CD90, CD166 (mesenchymal progenitor markers), Stro1, and Jagged1 (presumptive chondroprogenitor markers) at a more intense level when CCPs are under pathological conditions. Nevertheless, CCPs are a small population, and their properties and detailed biochemistry need further study.

### Ctsk^+^ Periosteal Mesenchymal Stem/Progenitor Cells

Bones contain discrete multipotent stem cell populations that can generate various lineages (Chan et al., 2015; Worthley et al., 2015; Debnath et al., 2018; Gao et al., 2019). Hence, the lack of genetic markers distinguishing periosteal mesenchyme from endosteal mesenchyme has been a critical limitation in identifying periosteal stem/progenitor cells. Intriguingly, Debnath et al. observed CTSK-mGFP cells in periosteal mesenchyme of *Ctsk-cre;mTmG* reporter mice on embryonic day 14.5 (E14.5), and these cells were detected in the endosteal marrow compartment as well on postnatal day 10. The CTSK-mGFP cells in the endosteal marrow compartment were confirmed to be osteoclasts by flow cytometry and co-staining for TRAP (Debnath et al., 2018; Han et al., 2019). The CTSK-mGFP cells in the periosteum manifested CD45^−^, TER119^−^, and CD31^−^ (hereafter Lin^−^). Fractionation of CTSK-mGFP mesenchymal cells using multicolor flow cytometry showed three distinct populations, including PSCs (CD200^+^ and CD105^−^), periosteal progenitor 1 (PP1) (CD200^−^ and CD105^−^), and periosteal progenitor 2 (PP2) (CD105^+^, CD200_variable_), where PSCs exhibited self-renewal ability and multipotency of mesenchymal stem cells, and ranked the most dedifferentiation (Table 1). Moreover, a subpopulation of CTSK-mGFP cells also expressed Gremlin1 and Nestin, but the CTSK-mGFP cells did not display markers of hematopoietic mesenchymal cells, such as LEPR, CD146, and CD140α (Sacchetti et al., 2007; Morikawa et al., 2009; Pinho et al., 2013; Zhou et al., 2014; Debnath et al., 2018). Compared with non-Ctsk MSCs (Lin^−^, 6C3^−^, THY^−^, CD200^+^, CD105^−^, and GFP^−^), PSCs mediated the intramembranous bone formation. In contrast, non-Ctsk MSCs processed endochondral ossification with hematopoietic recruitment and cartilage formation, although they expressed the same genetic markers of mesenchymal stem/progenitor cells, including Runx2 and Sox9 (Chan et al., 2009). PSCs were found in the sutures of the calvarium, while CTSK-mGFP PP1, PP2, THY^+^, CD146^+^, and SCA1^+^ cells appeared outside the sutures of the calvarium. Craniofacial bones are formed mainly through intramembranous ossification (Maruyama, 2019), thereby indicating that PSCs generate bones in the same way. In parallel, Han et al. further demonstrated that Ctsk could label periosteal mesenchyme (Han et al., 2019). Rosa26-Ai9 reporter mice showed that Ctsk-Ai9^+^ cells expressed the common stem cell markers, including Sca1, CD24, CD44, CD49f, and CD146. Moreover, alkaline phosphatase (ALP), Alcian blue, and oil red O staining indicated the multidirectional differentiation ability of the periosteal Ctsk^+^ cells, which was consistent with the observations of Debnath et al.

**TABLE 1:** Genetic markers of Ctsk-labeled cells and related diseases in the skeletal system.

Ctsk-Labeled cells	Genetic markers	Related diseases	Potential therapeutic targets	References
Osteoclasts	TRAP^+^	Osteoporosis	Cathepsin K inhibitor	Drake et al. (1996) Brömme et al. (2016) McClung et al. (2019)
Chondrocytes and fibroblast-like synoviocytes	Not mentioned	Osteoarthritis (OA) and rheumatoid arthritis (RA)	Cathepsin K inhibitor	Morko et al. (2004) Kozawa et al. (2016)
Ctsk + chondroid progenitors (CCPs)	CD44^+^, CD90^+^, CD166^+^, Stro1^+^, and Jagged1^+^	Metachondromatosis (MC)	Smoothened inhibitor	Karlsson et al. (2009) Yang et al. (2013) Usami et al. (2019)
Periosteal mesenchymal stem cells (PSCs)	CD200^+^ and CD105^−^	Osteosarcoma and bone regeneration	mTORC1 inhibition and cathepsin K inhibitor	Gentile et al. (2014) Debnath et al. (2018) Han et al. (2019)
Periosteal progenitor 1 (PP1)	CD200^−^ and CD105^−^			
Periosteal progenitor 2 (PP2)	CD105^+^ and CD200_variable_			
Tendon-derived progenitor cells (TDPCs)	Scx^+^, CD44^+^, CD105^+^, Nestin^+^, and Sca1^+^	Heterotopic ossification (HO)	JQ1	Bi et al. (2007) Feng et al. (2020)

### Ctsk^+^ TDPCs

*In vitro* TDPCs are widely studied, thereby showing excellent self-renewal capacity and multipotency (Bi et al., 2007) of TDPCs. However, their *in vivo* studies are rare. Notably, Ai9 reporter mice showed that *Ctsk-Cre* could label a subpopulation of TDPCs (Feng et al., 2020). These Ctsk-cre–expressing cells were detected in most cells within the Achilles tendon, quadriceps tendon, and tendinous insertions of the patella *in vivo*. They also expressed tendon-related marker *Scx* after crossbreeding with *ScxGFP* mice. Ctsk^+^Scx^+^ cells classified using fluorescence-activated cell sorting were expressed as CD44, CD105, Nestin, Sca1, and other genetic markers of stem/progenitor cells, including CD24 and CD200. In addition, Ctsk^+^Scx^+^ cells exhibited higher differentiation potential into osteoblasts, chondrocytes, and adipocytes than Ctsk^+^Scx^–^ tendon-derived cells, while neither Ctsk^–^Scx^–^ nor Ctsk^–^Scx^+^ tendon-derived cells lacked the capacity.

## Role of Ctsk^+^ Cells in Pathogenesis and Therapeutic Strategies

**FIGURE 2:**
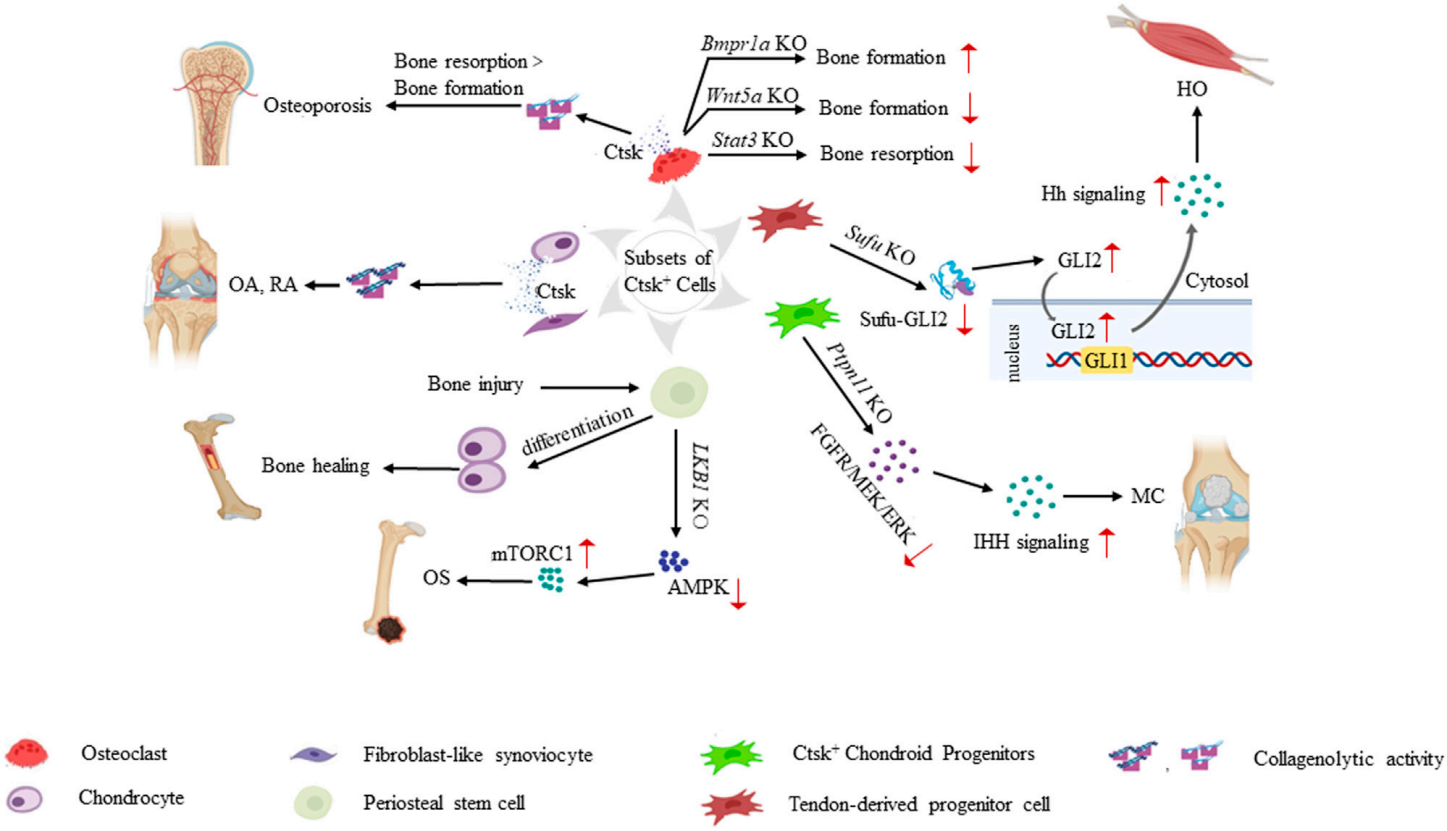
Role of Ctsk^+^ cells in the pathogenesis of various skeletal system diseases. Ctsk^+^ cells help determine the origin cells of some musculoskeletal system-related diseases. Increased secretion of Ctsk from mature osteoclasts and chondrocytes leads to enhanced collagenolytic activity and subsequently contributes to osteoporosis, OA, and RA. Knockouts of some key genes, such as *Bmpr1a*, *Wnt5a*, and *Stat3* in Ctsk^+^ osteoclasts break the balance of bone formation and resorption. In Ctsk^+^ mesenchymal stem/progenitor cells, Ctsk serves as a genetic marker and helps us deeply understand the property and function of mesenchymal stem/progenitor cells. PSCs play a significant role in intramembranous bone formation under physiological conditions. They differentiate into chondrocytes after bone injury, thereby participating in endochondral bone formation to promote bone healing. PSCs also serve as pathological precursors in osteogenic tumors through the LKB1/AMPK/mTORC1 signaling pathways. Ptpn11 depletion in CCPs leads to MC via the ERK and Ihh signaling pathways. Ctsk^+^ TDPCs with targeted *Sufu* deletion result in heterotopic HO via Hh pathway activation. Translocation of GLI2 from the cytoplasm to the nucleus transactivates the *GLI1* promoter. Increased Gli1/Gli2 in Ctsk^+^ TDPCs enhances the Hh signaling activity and promotes HO progression.

Many studies have worked on the role of Ctsk-expressing cells in bone remodeling and cellular regulation (especially progenitor/stem cells) (Figure 2). These studies suggest that Ctsk-labeled cells may be further investigated to understand the pathogenesis of various skeletal disorders and could provide an attractive therapeutic target in clinical practice.

### Ctsk^+^ Osteoclasts and Osteoporosis

Ctsk-Cre mice have been widely used to study the functional properties of osteoclasts in preclinical models of osteoporosis. BMP signal in osteoblasts is elucidated to promote bone formation via the canonical Wnt signaling pathway (Kamiya et al., 2008a; Kamiya et al., 2008b; Okamoto et al., 2011). Tamoxifen-induced osteoblast-specific ablation of *Bmpr1a* decreases the osteoclast number and bone resorption activity in mice. Conversely, conditional knockout of *Bmpr1a* in differentiated osteoclasts in Ctsk-Cre mouse models enhances osteogenesis. Hence, Ctsk^+^ osteoclasts can interfere with the activities of osteoblasts in a BMPR1A signaling-dependent manner. A previous study found that Wnt5a secreted by osteoblasts activated Rank expression to stimulate osteoclastogenesis (Maeda et al., 2012). Recently, *Wnt5a* loss-of-function in Ctsk^+^ osteoclast was tested by intercrossing *Wnt5a* ^*fl/f*^ and Ctsk-Cre mouse lines. *Ctsk-Cre;Wnt5a* ^*fl/f*^ mice exhibited reduced trabecular and cortical bone without osteoclast elevation (Roberts et al., 2020). *Stat3* participated in maintaining bone homeostasis in osteoblasts (Zhou et al., 2011). Furthermore, *Stat3* deletion in bone marrow macrophages was reported to reduce NFATc1 expression and decrease osteoclast differentiation, and *Stat3* deletion in osteoclasts led to a decreased osteoclast activity (Yang et al., 2019; Yang et al., 2020). These molecules exerted exactly opposing effects on bone remodeling as they were derived from different types of cells. Mechanistically, the exact role of Ctsk in osteoclasts was elucidated by Lotinun et al. (Lotinun et al., 2013). They used a specific conditional knockout of CD11b-Cre to exclusively target the monocyte-macrophage lineage to generate *CD11b-Cre;Ctsk* ^*fl/fl*^. Ctsk deleted in osteoclasts revealed an increased bone formation rate, while *cathepsin K* knockout in Osx-Cre mice specifically targeted to osteoblasts did not exhibit any skeletal phenotype.

### Ctsk^+^ Chondrocytes and Synovial Cells and Osteoarthritis and Rheumatoid Arthritis

Progressive cartilage damage is the key process that contributes to osteoarthritis (OA) and rheumatoid arthritis (RA) (Pap and Korb-Pap, 2015). Ctsk oligomerized with chondroitin sulfate, particularly chondroitin-4-sulfate molecules, was likely to degrade collagen Ⅱ (Li et al., 2000; Li et al., 2002). Moreover, *cathepsin K* mRNA expression increases in the articular chondrocytes near the matrix destruction of the transgenic osteoarthritic model at the onset of cartilage degeneration. In contrast, only a tiny minority of chondrocytes were observed expressing Ctsk in the control mice. With age, Ctsk also increased in the control group with developed OA (Morko et al., 2004). Accordingly, *Ctsk* ^*−/−*^ mice were used to investigate the role of Ctsk in OA progression (Li et al., 2006). The study showed that *Ctsk* deficiency slowed down OA development, particularly in the early to intermediate stages compared with *Ctsk* ^*+/+*^ mice. *Ctsk* deficiency could cause delayed remodeling of subchondral and calcified cartilage, which protects the articular cartilage (Soki et al., 2018). There existed a positive correlation between the severity of OA and the expression of Ctsk in chondrocytes (Konttinen et al., 2002). Consistently, Ctsk was expressed in chondrocytes in humans, and its collagenolytic activity was augmented in OA patients (Kozawa et al., 2016). Interestingly, a recent study demonstrated that mechanical stress loading enhanced *Ctsk* expression of protein in human chondrocytic HCS-2/8 cells, which was suppressed by hyaluronan (Suzuki et al., 2020). Using the pharmacological approach, current treatments of OA act mainly on symptom improvement without slowing down the OA development.

Furthermore, RA-associated joint destruction is caused by synovial fibroblast-mediated collagen degradation. Cartilage degradation started at the most superficial layer in the monkey collagen-induced arthritis (CIA) model (Tanaka et al., 2016). Rheumatoid arthritis and periodontitis share many pathological features; Ctsk deletion in the RA and periodontitis mice models ameliorates inflammation and bone erosion in both of them owing to its shared osteoimmune role (Hao et al., 2015). Ctsk is activated by immune cells through the TLR4, 5, and 9 signaling pathways, and the inhibition of Ctsk reduces TLR9 expression and downregulates the autophagy signaling pathway (Wei et al., 2020). In addition, in the CIA mouse model, the inhibition of Ctsks alleviates bone erosion, cartilage degradation, and inflammation at joints and enhances bone strength (Svelander et al., 2009; Yamashita et al., 2018).

### Ctsk^+^ Chondroid Progenitor Cells and Cartilaginous Tumor

Metachondromatosis (MC), a rare hereditary disease of the skeletal system, is characterized by multiple osteochondroma and enchondroma. Hereditary multiple osteochondroma is a benign tumor that grows outward from the metaphyses of long bones, and enchondroma is a benign cartilaginous tumor that grows endophytic to the bone (Pannier and Legeai-Mallet, 2008). Proteintyrosine phosphatase nonreceptor type 11 (*ptpn11*) encodes the nonreceptor proteintyrosine phosphatase SHP2. Targeted deleting of *Ptpn11* in chondrocytes contributed to the MC phenotype (Kim et al., 2014). In addition, *Ptpn11* depletion in *Ctsk*-expressing cells caused bone phenotypes consistent with MC using *Ctsk-cre;Ptpn11* ^*fl/fl*^ (Ctsk-KO) transgenic mice (the Ctsk promoter is active only in mature osteoclasts). In contrast, mild osteopetrosis presented in *lysozyme M-cre;Ptpn11* ^*fl/fl*^ mice (the LysM promoter is active in monocytes, macrophages, and osteoclast precursors) (Faust et al., 2000; Yang et al., 2013). Lineage tracing indicated that Ctsk^+^ perichondral cells within “Groove of Ranvier” are labeled pathological cells. Notably, YFP^+^ cells had entered and differentiated into ectopic cartilaginous tissues by postnatal week 2 in Ctsk-KO/YFP reporter mice. The CCPs differentiate into chondrocytes at different stages of development in lesions using cell morphology and Col2α1 and Col10α1 immunostaining, including proliferating, prehypertrophic, and hypertrophic chondrocytes. Ptpn11 is required for fibroblast growth factor/mitogen-activated protein kinase/extracellular signal-regulated kinase (FGFR/MEK/ERK)-dependent pathways in various growths and development-related signaling pathways (Grossmann et al., 2010). The *Ctsk-cre;Ptpn11* ^*fl/fl*^ mice showed significantly decreased ERK expressions. Smoothened inhibitor PF-04449913 treatment is believed to suppress the Ihh target gene expression and reduce the number of exostoses. It slows down the MC progression in *Ctsk-cre;Ptpn11* ^*fl/fl*^ mice. Almost all chondroid tumor cells were YFP^+^ in *Ctsk-KO/YFP* reporter mice, thereby indicating that specific genetic blockade of CCPs could serve as pathological cells that contribute to the pathogenesis of MC, although CCPs are rare. Liver kinase b1 (*Lkb1*) is a master serine/threonine kinase that plays a crucial role in energy homeostasis and cell growth (Shaw, 2009). *Lkb1* deficiency in periosteum-derived Ctsk^+^ cells may cause an osteogenic tumor-like phenotype (Han et al., 2019). However, the loss of *Lkb1* in chondrocytes contributes to enchondroma-like tumors (Lai et al., 2013). Accordingly, disruptions of *Lkb1* in specific types of cells lead to different kinds of tumor-like phenotypes via respective signaling pathways. Further experimental studies on the detailed properties of CCPs are needed to understand how these crucial regulators mediate molecular actions in CCPs and seek more novel attractive targets for therapeutic strategies for cartilaginous diseases.

### Ctsk^+^ Periosteal Mesenchymal Progenitors and Osteogenic Tumor

Osteosarcoma (OS) is a malignant neoplasm with an unfavorable prognosis that primarily occurs in children and adolescents. Previous studies have reported that mesenchymal progenitors (Prx1-cre), osteoblast precursors (Osx-cre), and osteoblast-committed cells (Col1a1-Cre and OCN-Cre), which are of mesenchymal origin and osteogenic lineage, can function as the pathological precursors contributing to OS (Walkley et al., 2008; Calo et al., 2010; Abarrategi et al., 2016; Del Mare et al., 2016). The periosteum-derived Ctsk^+^ cells congruously acted with mesenchymal stem cells; therefore, the deletion of *LKB1* in Ctsk-expressing periosteal mesenchymal cells caused osteogenic tumor, as expected (Han et al., 2019). This finding advanced knowledge of the pathogenesis of osteogenic tumors in a specific type of cell. It stimulated targeted therapeutic approaches for new strategies, although the exact role of Ctsk in these stem cell-like periosteal cells remained unknown. Liver kinase B1 is a tumor suppressor, and a decreased Lkb1 protein expression was detected in 41% of patients with osteosarcoma (Presneau et al., 2017). Lkb1 deficiency stimulates the proliferation and osteogenesis of Ctsk^+^ periosteal mesenchymal cells. In addition, mTORC1 activation observed in OS patients is the downstream of LKB1-dependent adenosine monophosphate (AMP) kinases (AMPKs). Simultaneously, knockout of Lkb1 in Prx1^+^ mesenchymal progenitors resulted in osteoid tumors transgressing the cortex and BM cavity. However, both the femur and tibia of *Prx1-cre;Lkb1* ^*fl/fl*^ mice displayed an enchondroma-like phenotype, which was different from the observation in *Ctsk-cre;Lkb1* ^*fl/fl*^ mice. This may be due to the Prx1^+^ and Ctsk^+^ mesenchymal progenitor distribution; Prx1^+^ mesenchymal progenitors reside in both the periosteum and bone marrow, and Ctsk^+^ mesenchymal progenitors are seen only in the periosteum (Wang et al., 2016; Han et al., 2019). Conditional knockout of *Osterix* (*Osx*) in *Ctsk-Cre* mice produces a marginal effect on endosteal bone mass and significantly decreases the periosteal bone formation (Debnath et al., 2018). These observations defined periosteum-derived Ctsk^+^ cells as a novel subpopulation of mesenchymal progenitors. Inspiringly, mTORC1 inhibition used in *Ctsk-cre;Lkb1* ^*fl/fl*^ mice may slow down tumorigenesis progression. In the context of unsatisfactory traditional chemotherapy treatment, LKB1/AMPK/mTORC1 signaling provides a novel targeted therapeutic approach for treating osteosarcoma.

### Ctsk^+^ Periosteal Cells and Bone Regeneration

The periosteum is a reservoir of quiescent mesenchymal progenitor cells that facilitate endochondral bone formation to promote bone regeneration after bone injury (Colnot, 2009). The available evidence from preclinical studies has demonstrated that a subset of Ctsk-positive (PSCs) participate in intramembranous bone formation to maintain bone homeostasis under physiological conditions. However, when the bone is damaged, PSCs differentiate into chondrocytes and participate in endochondral bone formation to promote fracture healing. Ctsk^+^ PSCs displayed more robust proliferation and osteogenic differentiation ability than non-Ctsk^+^ PSCs after fracture (Debnath et al., 2018). Injuries stimulate the proliferation and osteogenic differentiation of PSCs. Periostin, expressed explicitly in the periosteum, creates an osteogenic microenvironment (Horiuchi et al., 1999). Bonnet et al. described periostin as the substrate for degradation by Ctsk and Ctsk inhibitors or deletion stimulated periostin and β-catenin expression (Bonnet et al., 2017). Deleting Ctsk also increased TRAP^+^ mononuclear cells and platelet-derived growth factor (PDGF)-BB secretion (Chen et al., 2007; Xie et al., 2014). PDGF-BB is necessary for Nestin^+^ and LepR^+^ periosteum-derived cells to migrate to the periosteal surface for bone formation and periosteum homeostasis (Gao et al., 2019). Periosteal osteoclast precursors (OCPs), instead of bone marrow osteoclast precursors, were significantly upregulated in the *Ctsk* ^−/−^ periosteum upon fracture (Walia et al., 2018). Its secretion of PDGF-BB and S1P in the periosteum stimulated osteogenic differentiation, thereby intensifying bone mineralization and strength. Meanwhile, PDGF-BB binding to the PDGF receptor may trigger the PI3K/Akt/FAK signaling pathway and promote angiogenesis coupling with osteogenesis (Gentile et al., 2014; Xie et al., 2014). However, a study indicated that OCPs from *Ctsk* ^*−/−*^ mice were disabled to migrate and engraft in the bone callus in fractured mice (Jacome-Galarza et al., 2014).

### Ctsk^+^ TDPCs and Heterotopic Ossification

Heterotopic ossification (HO) is an aberrant phenomenon where the bone cells surround soft tissues, such as muscles, tendons, ligaments, and joints (Xu et al., 2018). Scx^+^ progenitor lineages are responsible for spontaneous ligament, tendon, joint, and trauma-induced HO (Dey et al., 2016; Agarwal et al., 2017). Recently, Ctsk^+^ TDPCs have been identified as a novel subtype of progenitors contributing to HO (Feng et al., 2020). The Ihh signaling activation in Ctsk-Cre cells produces MC (Yang et al., 2013). However, a disruption in the suppressor of fused (*Sufu*) in Ctsk-expressing cells, a suppressor regulating the Hh pathway, did not generate the presumptive phenotype. Instead, pathological bone formation is present in ligaments, tendons, joints, and other soft tissues. Zinc finger transcription factors GLI1 and GLI2 are critical in Hh signaling, while Sufu inhibits the translocation of GLI2 from the cytoplasm to the nucleus to transactivate the *GLI1* promoter (Varjosalo and Taipale, 2008). It is encouraging to learn that Gli1/Gli2 deficiency in Ctsk^+^ TDPCs disrupted the Hh signaling stability and ameliorated HO progression, thereby providing the rationale for HO clinical treatment as an attractive therapeutic target. The expressed Ctsk^+^ cells increased the chondrogenic differentiation marker *COLII* and the osteogenic differentiation markers *Opn*, *Ocn*, and *Alp*. However, they decreased the tendon-related genes *Scx*, *Mkx*, and *Tnmd*, thereby suggesting that Ctsk^+^ TDPCs could differentiate into chondrocytes and osteoblasts; the Hh signaling activation changed the fate of Ctsk^+^ TDPCs. Gant61 was used to treat Hh-overactive tumors in mice (Lauth et al., 2007; Fu et al., 2013), but it failed to ameliorate HO caused by *Sufu* deficiency in Ctsk^+^ TDPCs. Feng et al. found a small-molecule inhibitor, JQ1, as the pharmacological inhibitor of Hh signaling. JQ1 downregulated Hh signaling in Ctsk^+^ TDPCs from *Ctsk-cre;Sufu* ^*fl/fl*^ mice. Disappointedly, no CreERT2 mouse line was designed to observe the specific time when the cells were marked and to determine the exact role of Ctsk^+^ lineage cells in trauma-induced HO. Cumulatively, the molecular mechanisms and mouse models remain to be explored further.

### Cathepsin K Inhibitor in Translational Applications

Ctsk inhibitors can treat skeletal diseases, such as osteoporosis, osteoarthritis, RA, and bone fracture, with odanacatib being the most well-known inhibitor. Previous studies have shown that odanacatib has therapeutic effects on osteoporosis and bone fracture in postmenopausal women (Bone et al., 2010; Eisman et al., 2011; Langdahl et al., 2012; Brixen et al., 2013; Cheung et al., 2014; Rizzoli et al., 2016). However, odanacatib increased the risk of stroke in phase III clinical trials; therefore, its use was discontinued (McClung et al., 2019). There may be overlapping disease pathways in the bone and cardiovascular biology, which should be further researched to guide therapies for these two diseases. The other two Ctsk inhibitors that progressed to clinical studies include ONO-5334 (Phase 1 and 2 studies) and MIV-711 (Phase 1 and 2 studies). ONO-5334 effectively elevated the BMD in a phase II trial for postmenopausal osteoporosis (Tanaka et al., 2017). It also prevented joint destruction in the monkey CIA model (Yamada et al., 2019). There are no obvious safety implications in the phase II study for osteoporosis; thus, ONO-5334 can be a potential drug for preventing joint destruction in RA patients. Furthermore, MIV-711, as a novel selective inhibitor, alleviated cartilage degradation in a phase-IIa trial, with a marginal effect on pain relief (Conaghan et al., 2020). In general, a Ctsk inhibitor is still a researchable hotspot for osteoporosis.

## Conclusion

Abundant evidence demonstrates that Ctsk is a pivotal lysosomal cysteine protease that participates in the physiological process of bone resorption. Furthermore, Ctsk, a biomarker of some subsets of stem/progenitor cells, assists in determining the origin cells of skeletal diseases. To date, the pathogenesis and promising therapeutic values of these diseases in Ctsk^+^ stem/progenitor cells are scarce. The precise function of Ctsk secreted by these stem/progenitor cells remains unclear. One study showed that as a metastasis-related protein upregulated by the imbalance of intestinal microbiota (Li et al., 2019), Ctsk enhances the migration and motility of colorectal cancer cells. We may speculate that Ctsk promotes the migration of Ctsk^+^ stem/progenitor cells in bone homeostasis and fracture healing. Hence, the true nature of Ctsk^+^ cells in the *in vivo* models is the target of extensive studies. Data recorded from these genetic models are precious but require scrutiny and comprehensive analysis of their pitfalls. The role of Ctsk in some osseous diseases is well proven, but its role in other types of bone tumors, such as osteomalacia and rachitis, is unknown. Future studies with improved *in vivo* models would help define the nature of Ctsk^+^ cells and seek more salutary targets for the clinical treatment of these diseases.

## References

[B1] Abarrategi A. Tornin J. Martinez-Cruzado L. Hamilton A. Martinez-Campos E. Rodrigo J. P. (2016). Osteosarcoma: Cells-Of-Origin, Cancer Stem Cells, and Targeted Therapies. Stem Cells Int. 2016, 3631764. 10.1155/2016/3631764 27366153 PMC4913005

[B2] Agarwal S. Loder S. J. Cholok D. Peterson J. Li J. Breuler C. (2017). Scleraxis-Lineage Cells Contribute to Ectopic Bone Formation in Muscle and Tendon. Stem Cells 35 (3), 705–710. 10.1002/stem.2515 27862618 PMC5529170

[B3] Bernstein H.-G. Bukowska A. Dobrowolny H. Bogerts B. Lendeckel U. (2007). Cathepsin K and Schizophrenia. Synapse 61 (4), 252–253. 10.1002/syn.20358 17230547

[B4] Bi Y. Ehirchiou D. Kilts T. M. Inkson C. A. Embree M. C. Sonoyama W. (2007). Identification of Tendon Stem/progenitor Cells and the Role of the Extracellular Matrix in Their Niche. Nat. Med. 13 (10), 1219–1227. 10.1038/nm1630 17828274

[B5] Bone H. G. McClung M. R. Roux C. Recker R. R. Eisman J. A. Verbruggen N. (2010). Odanacatib, a Cathepsin-K Inhibitor for Osteoporosis: a Two-Year Study in Postmenopausal Women with Low Bone Density. J. Bone Min. Res. 25 (5), 937–947. 10.1359/jbmr.091035 19874198

[B6] Bonnet N. Brun J. Rousseau J.-C. Duong L. T. Ferrari S. L. (2017). Cathepsin K Controls Cortical Bone Formation by Degrading Periostin. J. Bone Min. Res. 32 (7), 1432–1441. 10.1002/jbmr.3136 28322464

[B7] Brixen K. Chapurlat R. Cheung A. M. Keaveny T. M. Fuerst T. Engelke K. (2013). Bone Density, Turnover, and Estimated Strength in Postmenopausal Women Treated with Odanacatib: a Randomized Trial. J. Clin. Endocrinol. Metab. 98 (2), 571–580. 10.1210/jc.2012-2972 23337728

[B8] Brömme D. Panwar P. Turan S. (2016). Cathepsin K Osteoporosis Trials, Pycnodysostosis and Mouse Deficiency Models: Commonalities and Differences. Expert Opin. Drug Discov. 11 (5), 457–472. 10.1517/17460441.2016.1160884 27001692

[B9] Brubaker K. Vessella R. True L. Thomas R. Corey E. (2003). Cathepsin K mRNA and Protein Expression in Prostate Cancer Progression. J. Bone Min. Res. 18 (2), 222–230. 10.1359/jbmr.2003.18.2.222 12568399

[B10] Bühling F. Gerber A. Häckel C. Krüger S. Köhnlein T. Brömme D. (1999). Expression of Cathepsin K in Lung Epithelial Cells. Am. J. Respir. Cell Mol. Biol. 20 (4), 612–619. 10.1165/ajrcmb.20.4.3405 10100992

[B11] Bühling F. Reisenauer A. Gerber A. Krüger S. Weber E. Brömme D. (2001). Cathepsin K - a Marker of Macrophage Differentiation? J. Pathol. 195 (3), 375–382. 10.1002/path.959 11673837

[B12] Bühling F. Röcken C. Brasch F. Hartig R. Yasuda Y. Saftig P. (2004). Pivotal Role of Cathepsin K in Lung Fibrosis. Am. J. Pathology 164 (6), 2203–2216. 10.1016/s0002-9440(10)63777-7 PMC1615770 15161653

[B13] Calo E. Quintero-Estades J. A. Danielian P. S. Nedelcu S. Berman S. D. Lees J. A. (2010). Rb Regulates Fate Choice and Lineage Commitment *In Vivo* . Nature 466 (7310), 1110–1114. 10.1038/nature09264 20686481 PMC2933655

[B14] Chan C. K. Seo E. Y. Chen J. Y. Lo D. McArdle A. Sinha R. (2015). Identification and Specification of the Mouse Skeletal Stem Cell. Cell 160 (1-2), 285–298. 10.1016/j.cell.2014.12.002 25594184 PMC4297645

[B15] Chan C. K. F. Chen C.-C. Luppen C. A. Kim J.-B. DeBoer A. T. Wei K. (2009). Endochondral Ossification Is Required for Haematopoietic Stem-Cell Niche Formation. Nature 457 (7228), 490–494. 10.1038/nature07547 19078959 PMC2648141

[B16] Chatani M. Takano Y. Kudo A. (2011). Osteoclasts in Bone Modeling, as Revealed by *In Vivo* Imaging, Are Essential for Organogenesis in Fish. Dev. Biol. 360 (1), 96–109. 10.1016/j.ydbio.2011.09.013 21963458

[B17] Chen W. Yang S. Abe Y. Li M. Wang Y. Shao J. (2007). Novel Pycnodysostosis Mouse Model Uncovers Cathepsin K Function as a Potential Regulator of Osteoclast Apoptosis and Senescence. Hum. Mol. Genet. 16 (4), 410–423. 10.1093/hmg/ddl474 17210673 PMC3578583

[B18] Cheung A. M. Majumdar S. Brixen K. Chapurlat R. Fuerst T. Engelke K. (2014). Effects of Odanacatib on the Radius and Tibia of Postmenopausal Women: Improvements in Bone Geometry, Microarchitecture, and Estimated Bone Strength. J. Bone Min. Res. 29 (8), 1786–1794. 10.1002/jbmr.2194 24643905

[B19] Chiellini C. Costa M. Novelli S. E. Amri E.-Z. Benzi L. Bertacca A. (2003). Identification of Cathepsin K as a Novel Marker of Adiposity in White Adipose Tissue. J. Cell. Physiol. 195 (2), 309–321. 10.1002/jcp.10253 12652657

[B20] Chiu W. S. M. McManus J. F. Notini A. J. Cassady A. I. Zajac J. D. Davey R. A. (2004). Transgenic Mice that Express Cre Recombinase in Osteoclasts. Genesis 39 (3), 178–185. 10.1002/gene.20041 15282744

[B21] Colnot C. (2009). Skeletal Cell Fate Decisions within Periosteum and Bone Marrow during Bone Regeneration. J. Bone Mineral Res. 24 (2), 274–282. 10.1359/jbmr.081003 PMC3276357 18847330

[B22] Conaghan P. G. Bowes M. A. Kingsbury S. R. Brett A. Guillard G. Rizoska B. (2020). Disease-Modifying Effects of a Novel Cathepsin K Inhibitor in Osteoarthritis. Ann. Intern Med. 172 (2), 86–95. 10.7326/m19-0675 31887743

[B23] Debnath S. Yallowitz A. R. McCormick J. Lalani S. Zhang T. Xu R. (2018). Discovery of a Periosteal Stem Cell Mediating Intramembranous Bone Formation. Nature 562 (7725), 133–139. 10.1038/s41586-018-0554-8 30250253 PMC6193396

[B24] Del Mare S. Husanie H. Iancu O. Abu-Odeh M. Evangelou K. Lovat F. (2016). WWOX and P53 Dysregulation Synergize to Drive the Development of Osteosarcoma. Cancer Res. 76 (20), 6107–6117. 10.1158/0008-5472.can-16-0621 27550453 PMC5146760

[B25] Dey D. Bagarova J. Hatsell S. J. Armstrong K. A. Huang L. Ermann J. (2016). Two Tissue-Resident Progenitor Lineages Drive Distinct Phenotypes of Heterotopic Ossification. Sci. Transl. Med. 8 (366), 366ra163. 10.1126/scitranslmed.aaf1090 PMC6407419 27881824

[B26] Drake F. H. Dodds R. A. James I. E. Connor J. R. Debouck C. Richardson S. (1996). Cathepsin K, but Not Cathepsins B, L, or S, Is Abundantly Expressed in Human Osteoclasts. J. Biol. Chem. 271 (21), 12511–12516. 10.1074/jbc.271.21.12511 8647859

[B27] Eisman J. A. Bone H. G. Hosking D. J. McClung M. R. Reid I. R. Rizzoli R. (2011). Odanacatib in the Treatment of Postmenopausal Women with Low Bone Mineral Density: Three-Year Continued Therapy and Resolution of Effect. J. Bone Min. Res. 26 (2), 242–251. 10.1002/jbmr.212 20740685

[B28] Fan X. Wang C. Song X. Liu H. Li X. Zhang Y. (2018). Elevated Cathepsin K Potentiates Metastasis of Epithelial Ovarian Cancer. Histol. Histopathol. 33 (7), 673–680. 10.14670/HH-11-960 29303207

[B29] Faust N. Varas F. Kelly L. M. Heck S. Graf T. (2000). Insertion of Enhanced Green Fluorescent Protein into the Lysozyme Gene Creates Mice with Green Fluorescent Granulocytes and Macrophages. Blood 96 (2), 719–726. 10.1182/blood.v96.2.719 10887140

[B30] Feng H. Xing W. Han Y. Sun J. Kong M. Gao B. (2020). Tendon-derived Cathepsin K-Expressing Progenitor Cells Activate Hedgehog Signaling to Drive Heterotopic Ossification. J. Clin. Invest. 130 (12), 6354–6365. 10.1172/jci132518 32853181 PMC7685727

[B31] Fenichel I. Evron Z. Nevo Z. (2006). The Perichondrial Ring as a Reservoir for Precartilaginous Cells. *In Vivo* Model in Young Chicks' Epiphysis. Int. Orthop. (SICOT) 30 (5), 353–356. 10.1007/s00264-006-0082-2 PMC3172782 16652202

[B32] Fu J. Rodova M. Roy S. K. Sharma J. Singh K. P. Srivastava R. K. (2013). GANT-61 Inhibits Pancreatic Cancer Stem Cell Growth *In Vitro* and in NOD/SCID/IL2R Gamma Null Mice Xenograft. Cancer Lett. 330 (1), 22–32. 10.1016/j.canlet.2012.11.018 23200667 PMC4153855

[B33] Gao B. Deng R. Chai Y. Chen H. Hu B. Wang X. (2019). Macrophage-lineage TRAP+ Cells Recruit Periosteum-Derived Cells for Periosteal Osteogenesis and Regeneration. J. Clin. Invest. 129 (6), 2578–2594. 10.1172/jci98857 30946695 PMC6538344

[B34] Gao L.-h. Li S.-s. Yue H. Zhang Z.-l. (2020). Associations of Serum Cathepsin K and Polymorphisms in CTSK Gene with Bone Mineral Density and Bone Metabolism Markers in Postmenopausal Chinese Women. Front. Endocrinol. 11, 48. 10.3389/fendo.2020.00048 PMC7031211 32117071

[B35] Garnero P. Borel O. Byrjalsen I. Ferreras M. Drake F. H. McQueney M. S. (1998). The Collagenolytic Activity of Cathepsin K Is Unique Among Mammalian Proteinases. J. Biol. Chem. 273 (48), 32347–32352. 10.1074/jbc.273.48.32347 9822715

[B36] Gelb B. D. Moissoglu K. Zhang J. Martignetti J. A. Brömme D. Desnick R. J. (1996). Cathepsin K: Isolation and Characterization of the Murine cDNA and Genomic Sequence, the Homologue of the Human Pycnodysostosis Gene. Biochem. Mol. Med. 59 (2), 200–206. 10.1006/bmme.1996.0088 8986645

[B37] Gentile M. A. Soung D. Y. Horrell C. Samadfam R. Drissi H. Duong L. T. (2014). Increased Fracture Callus Mineralization and Strength in Cathepsin K Knockout Mice. Bone 66, 72–81. 10.1016/j.bone.2014.04.032 24928497

[B38] Gistelinck C. Gioia R. Gagliardi A. Tonelli F. Marchese L. Bianchi L. (2016). Zebrafish Collagen Type I: Molecular and Biochemical Characterization of the Major Structural Protein in Bone and Skin. Sci. Rep. 6, 21540. 10.1038/srep21540 26876635 PMC4753508

[B39] Gowen M. Lazner F. Dodds R. Kapadia R. Feild J. Tavaria M. (1999). Cathepsin K Knockout Mice Develop Osteopetrosis Due to a Deficit in Matrix Degradation but Not Demineralization. J. Bone Min. Res. 14 (10), 1654–1663. 10.1359/jbmr.1999.14.10.1654 10491212

[B40] Grossmann K. S. Rosário M. Birchmeier C. Birchmeier W. (2010). The Tyrosine Phosphatase Shp2 in Development and Cancer. Adv. Cancer Res. 106, 53–89. 10.1016/s0065-230x(10)06002-1 20399956

[B41] Gu X. Peng Y. Zhao Y. Liang X. Tang Y. Liu J. (2019). A Novel Derivative of Artemisinin Inhibits Cell Proliferation and Metastasis via Down-Regulation of Cathepsin K in Breast Cancer. Eur. J. Pharmacol. 858, 172382. 10.1016/j.ejphar.2019.05.011 31112710

[B42] Guo J. Bot I. de Nooijer R. Hoffman S. J. Stroup G. B. Biessen E. A. (2009). Leucocyte Cathepsin K Affects Atherosclerotic Lesion Composition and Bone Mineral Density in Low-Density Lipoprotein Receptor Deficient Mice. Cardiovasc Res. 81 (2), 278–285. 10.1093/cvr/cvn311 19015136

[B43] Haeckel C. Krueger S. Buehling F. Broemme D. Franke K. Schuetze A. (1999). Expression of Cathepsin K in the Human Embryo and Fetus. Dev. Dyn. 216 (2), 89–95. 10.1002/(sici)1097-0177(199910)216:2<89::aid-dvdy1>3.0.co;2-9 10536050

[B44] Han Y. Feng H. Sun J. Liang X. Wang Z. Xing W. (2019). Lkb1 Deletion in Periosteal Mesenchymal Progenitors Induces Osteogenic Tumors through mTORC1 Activation. J. Clin. Invest. 129 (5), 1895–1909. 10.1172/jci124590 30830877 PMC6486357

[B45] Hao L. Zhu G. Lu Y. Wang M. Jules J. Zhou X. (2015). Deficiency of Cathepsin K Prevents Inflammation and Bone Erosion in Rheumatoid Arthritis and Periodontitis and Reveals its Shared Osteoimmune Role. FEBS Lett. 589 (12), 1331–1339. 10.1016/j.febslet.2015.04.008 25896020 PMC4623593

[B46] Horiuchi K. Amizuka N. Takeshita S. Takamatsu H. Katsuura M. Ozawa H. (1999). Identification and Characterization of a Novel Protein, Periostin, with Restricted Expression to Periosteum and Periodontal Ligament and Increased Expression by Transforming Growth Factor β. J. Bone Min. Res. 14 (7), 1239–1249. 10.1359/jbmr.1999.14.7.1239 10404027

[B47] Hou W.-S. Li Z. Gordon R. E. Chan K. Klein M. J. Levy R. (2001). Cathepsin K Is a Critical Protease in Synovial Fibroblast-Mediated Collagen Degradation. Am. J. Pathology 159 (6), 2167–2177. 10.1016/s0002-9440(10)63068-4 PMC1850593 11733367

[B48] Hussein H. Boyaka P. Dulin J. Russell D. Smanik L. Azab M. (2017). Cathepsin K Localizes to Equine Bone *In Vivo* and Inhibits Bone Marrow Stem and Progenitor Cells Differentiation *In Vitro* . J. Stem Cells Regen. Med. 13 (2), 45–53. 10.46582/jsrm.1302008 29391749 PMC5786646

[B49] Inaoka T. Bilbe G. Ishibashi O. Tezuka K. Kumegawa M. Kokubo T. (1995). Molecular Cloning of Human cDNA for Cathepsin K: Novel Cysteine Proteinase Predominantly Expressed in Bone. Biochem. Biophysical Res. Commun. 206 (1), 89–96. 10.1006/bbrc.1995.1013 7818555

[B50] Jacome-Galarza C. Soung D. Y. Adapala N. S. Pickarski M. Sanjay A. Duong L. T. (2014). Altered Hematopoietic Stem Cell and Osteoclast Precursor Frequency in Cathepsin K Null Mice. J. Cell. Biochem. 115 (8), 1449–1457. 10.1002/jcb.24801 24590570

[B51] Kafienah W. Brömme D. Buttle D. J. Croucher L. J. Hollander A. P. (1998). Human Cathepsin K Cleaves Native Type I and II Collagens at the N-Terminal End of the Triple Helix. Biochem. J. 331 (Pt 3), 727–732. 10.1042/bj3310727 9560298 PMC1219411

[B52] Kamiya N. Ye L. Kobayashi T. Lucas D. J. Mochida Y. Yamauchi M. (2008). Disruption of BMP Signaling in Osteoblasts through Type IA Receptor (BMPRIA) Increases Bone Mass*. J. Bone Mineral Res. 23 (12), 2007–2017. 10.1359/jbmr.080809 PMC2686924 18684091

[B53] Kamiya N. Ye L. Kobayashi T. Mochida Y. Yamauchi M. Kronenberg H. M. (2008). BMP Signaling Negatively Regulates Bone Mass through Sclerostin by Inhibiting the Canonical Wnt Pathway. Development 135 (22), 3801–3811. 10.1242/dev.025825 18927151 PMC2694443

[B54] Karlsson C. Thornemo M. Henriksson H. B. Lindahl A. (2009). Identification of a Stem Cell Niche in the Zone of Ranvier within the Knee Joint. J. Anat. 215 (3), 355–363. 10.1111/j.1469-7580.2009.01115.x 19563472 PMC2750766

[B55] Kim H. K. Feng G.-S. Chen D. King P. D. Kamiya N. (2014). Targeted Disruption ofShp2in Chondrocytes Leads to Metachondromatosis with Multiple Cartilaginous Protrusions. J. Bone Min. Res. 29 (3), 761–769. 10.1002/jbmr.2062 PMC4081537 23929766

[B56] Kiviranta R. Morko J. Alatalo S. L. NicAmhlaoibh R. Risteli J. Laitala-Leinonen T. (2005). Impaired Bone Resorption in Cathepsin K-Deficient Mice Is Partially Compensated for by Enhanced Osteoclastogenesis and Increased Expression of Other Proteases via an Increased RANKL/OPG Ratio. Bone 36 (1), 159–172. 10.1016/j.bone.2004.09.020 15664014

[B57] Konttinen Y. T. Mandelin J. Li T.-F. Salo J. Lassus J. Liljeström M. (2002). Acidic Cysteine Endoproteinase Cathepsin K in the Degeneration of the Superficial Articular Hyaline Cartilage in Osteoarthritis. Arthritis & Rheumatism 46 (4), 953–960. 10.1002/art.10185 11953972

[B58] Kozawa E. Cheng X. W. Urakawa H. Arai E. Yamada Y. Kitamura S. (2016). Increased Expression and Activation of Cathepsin K in Human Osteoarthritic Cartilage and Synovial Tissues. J. Orthop. Res. 34 (1), 127–134. 10.1002/jor.23005 26241216

[B59] Kozawa E. Nishida Y. Cheng X. W. Urakawa H. Arai E. Futamura N. (2012). Osteoarthritic Change Is Delayed in a Ctsk-Knockout Mouse Model of Osteoarthritis. Arthritis & Rheumatism 64 (2), 454–464. 10.1002/art.33398 21968827

[B60] Lai L. P. Lilley B. N. Sanes J. R. McMahon A. P. (2013). Lkb1/Stk11 Regulation of mTOR Signaling Controls the Transition of Chondrocyte Fates and Suppresses Skeletal Tumor Formation. Proc. Natl. Acad. Sci. U.S.A. 110 (48), 19450–19455. 10.1073/pnas.1309001110 24218567 PMC3845115

[B61] Langdahl B. Binkley N. Bone H. Gilchrist N. Resch H. Rodriguez Portales J. (2012). Odanacatib in the Treatment of Postmenopausal Women with Low Bone Mineral Density: Five Years of Continued Therapy in a Phase 2 Study. J. Bone Min. Res. 27 (11), 2251–2258. 10.1002/jbmr.1695 22777865

[B62] Lauth M. Bergström Å. Shimokawa T. Toftgård R. (2007). Inhibition of GLI-Mediated Transcription and Tumor Cell Growth by Small-Molecule Antagonists. Proc. Natl. Acad. Sci. U.S.A. 104 (20), 8455–8460. 10.1073/pnas.0609699104 17494766 PMC1866313

[B63] Li C. Y. Jepsen K. J. Majeska R. J. Zhang J. Ni R. Gelb B. D. (2006). Mice Lacking Cathepsin K Maintain Bone Remodeling but Develop Bone Fragility Despite High Bone Mass. J. Bone Min. Res. 21 (6), 865–875. 10.1359/jbmr.060313 16753017

[B64] Li R. Zhou R. Wang H. Li W. Pan M. Yao X. (2019). Gut Microbiota-Stimulated Cathepsin K Secretion Mediates TLR4-dependent M2 Macrophage Polarization and Promotes Tumor Metastasis in Colorectal Cancer. Cell Death Differ. 26 (11), 2447–2463. 10.1038/s41418-019-0312-y 30850734 PMC6889446

[B65] Li Z. Hou W.-S. Brömme D. (2000). Collagenolytic Activity of Cathepsin K Is Specifically Modulated by Cartilage-Resident Chondroitin Sulfates. Biochemistry 39 (3), 529–536. 10.1021/bi992251u 10642177

[B66] Li Z. Hou W.-S. Escalante-Torres C. R. Gelb B. D. Brömme D. (2002). Collagenase Activity of Cathepsin K Depends on Complex Formation with Chondroitin Sulfate. J. Biol. Chem. 277 (32), 28669–28676. 10.1074/jbc.m204004200 12039963

[B67] Littlewood-Evans A. J. Bilbe G. Bowler W. B. Farley D. Wlodarski B. Kokubo T. (1997). The Osteoclast-Associated Protease Cathepsin K Is Expressed in Human Breast Carcinoma. Cancer Res. 57 (23), 5386–5390. 9393764

[B68] Lotinun S. Kiviranta R. Matsubara T. Alzate J. A. Neff L. Lüth A. (2013). Osteoclast-specific Cathepsin K Deletion Stimulates S1P-dependent Bone Formation. J. Clin. Invest. 123 (2), 666–681. 10.1172/JCI64840 23321671 PMC3561821

[B69] Lotinun S. Ishihara Y. Nagano K. Kiviranta R. Carpentier V. T. Neff L. (2019). Cathepsin K-Deficient Osteocytes Prevent Lactation-Induced Bone Loss and Parathyroid Hormone Suppression. J. Clin. Invest. 129 (8), 3058–3071. 10.1172/jci122936 31112135 PMC6668688

[B70] Lutgens E. Lutgens S. P. M. Faber B. C. G. Heeneman S. Gijbels M. M. J. de Winther M. P. J. (2006). Disruption of the Cathepsin K Gene Reduces Atherosclerosis Progression and Induces Plaque Fibrosis but Accelerates Macrophage Foam Cell Formation. Circulation 113 (1), 98–107. 10.1161/circulationaha.105.561449 16365196

[B71] Maeda K. Kobayashi Y. Udagawa N. Uehara S. Ishihara A. Mizoguchi T. (2012). Wnt5a-Ror2 Signaling between Osteoblast-Lineage Cells and Osteoclast Precursors Enhances Osteoclastogenesis. Nat. Med. 18 (3), 405–412. 10.1038/nm.2653 22344299

[B72] Mandelin J. Hukkanen M. Li T.-F. Korhonen M. Liljeström M. Sillat T. (2006). Human Osteoblasts Produce Cathepsin K. Bone 38 (6), 769–777. 10.1016/j.bone.2005.10.017 16337236

[B73] Maruyama T. (2019). Stem Cells of the Suture Mesenchyme in Craniofacial Bone Development, Repair and Regeneration. Keio J. Med. 68 (2), 42. 10.2302/kjm.68-003-abst 31243185

[B74] McClung M. R. O'Donoghue M. L. Papapoulos S. E. Bone H. Langdahl B. Saag K. G. (2019). Odanacatib for the Treatment of Postmenopausal Osteoporosis: Results of the LOFT Multicentre, Randomised, Double-Blind, Placebo-Controlled Trial and LOFT Extension Study. Lancet Diabetes Endocrinol. 7 (12), 899–911. 10.1016/S2213-8587(19)30346-8 31676222

[B75] McLellan M. A. Rosenthal N. A. Pinto A. R. (2017). Cre-loxP-Mediated Recombination: General Principles and Experimental Considerations. Curr. Protoc. Mouse Biol. 7 (1), 1–12. 10.1002/cpmo.22 28252198

[B76] Morikawa S. Mabuchi Y. Kubota Y. Nagai Y. Niibe K. Hiratsu E. (2009). Prospective Identification, Isolation, and Systemic Transplantation of Multipotent Mesenchymal Stem Cells in Murine Bone Marrow. J. Exp. Med. 206 (11), 2483–2496. 10.1084/jem.20091046 19841085 PMC2768869

[B77] Morko J. P. Soderstrom M. Saamanen A. M. Salminen H. J. Vuorio E. I. (2004). Up Regulation of Cathepsin K Expression in Articular Chondrocytes in a Transgenic Mouse Model for Osteoarthritis. Ann. Rheumatic Dis. 63 (6), 649–655. 10.1136/ard.2002.004671 PMC1755014 15140771

[B78] Mort J. S. Beaudry F. Théroux K. Emmott A. A. Richard H. Fisher W. D. (2016). Early Cathepsin K Degradation of Type II Collagen *In Vitro* and *In Vivo* in Articular Cartilage. Osteoarthr. Cartil. 24 (8), 1461–1469. 10.1016/j.joca.2016.03.016 27049030

[B79] Munari E. Cima L. Massari F. Bertoldo F. Porcaro A. B. Caliò A. (2017). Cathepsin K Expression in Castration-Resistant Prostate Carcinoma: a Therapeutical Target for Patients at Risk for Bone Metastases. Int. J. Biol. Markers 32 (2), e243–e7. 10.5301/jbm.5000246 28085175

[B80] Okamoto M. Murai J. Imai Y. Ikegami D. Kamiya N. Kato S. (2011). Conditional Deletion of Bmpr1a in Differentiated Osteoclasts Increases Osteoblastic Bone Formation, Increasing Volume of Remodeling Bone in Mice. J. Bone Min. Res. 26 (10), 2511–2522. 10.1002/jbmr.477 21786321

[B81] Pannier S. Legeai-Mallet L. (2008). Hereditary Multiple Exostoses and Enchondromatosis. Best Pract. Res. Clin. Rheumatology 22 (1), 45–54. 10.1016/j.berh.2007.12.004 18328980

[B82] Pap T. Korb-Pap A. (2015). Cartilage Damage in Osteoarthritis and Rheumatoid Arthritis-Two Unequal Siblings. Nat. Rev. Rheumatol. 11 (10), 606–615. 10.1038/nrrheum.2015.95 26195338

[B83] Petricevic S. J. Pavlovic A. Capkun V. Becic K. Durdov M. G. (2017). Cathepsin K Expression in Melanoma Is Associated with Metastases. Histol. Histopathol. 32 (7), 711–716. 10.14670/HH-11-833 27709599

[B84] Pinho S. Lacombe J. Hanoun M. Mizoguchi T. Bruns I. Kunisaki Y. (2013). PDGFRα and CD51 Mark Human Nestin+ Sphere-Forming Mesenchymal Stem Cells Capable of Hematopoietic Progenitor Cell Expansion. J. Exp. Med. 210 (7), 1351–1367. 10.1084/jem.20122252 23776077 PMC3698522

[B85] Presneau N. Duhamel L. A. Ye H. Tirabosco R. Flanagan A. M. Eskandarpour M. (2017). Post-translational Regulation Contributes to the Loss of LKB1 Expression through SIRT1 Deacetylase in Osteosarcomas. Br. J. Cancer 117 (3), 398–408. 10.1038/bjc.2017.174 28632727 PMC5537492

[B86] Rizzoli R. Benhamou C.-L. Halse J. Miller P. D. Reid I. R. Rodríguez Portales J. A. (2016). Continuous Treatment with Odanacatib for up to 8 Years in Postmenopausal Women with Low Bone Mineral Density: a Phase 2 Study. Osteoporos. Int. 27 (6), 2099–2107. 10.1007/s00198-016-3503-0 26879200

[B87] Roberts J. L. Liu G. Paglia D. N. Kinter C. W. Fernandes L. M. Lorenzo J. (2020). Deletion of Wnt5a in Osteoclasts Results in Bone Loss through Decreased Bone Formation. Ann. N.Y. Acad. Sci. 1463 (1), 45–59. 10.1111/nyas.14293 31919867

[B88] Sacchetti B. Funari A. Michienzi S. Di Cesare S. Piersanti S. Saggio I. (2007). Self-renewing Osteoprogenitors in Bone Marrow Sinusoids Can Organize a Hematopoietic Microenvironment. Cell 131 (2), 324–336. 10.1016/j.cell.2007.08.025 17956733

[B89] Saftig P. Hunziker E. Wehmeyer O. Jones S. Boyde A. Rommerskirch W. (1998). Impaired Osteoclastic Bone Resorption Leads to Osteopetrosis in Cathepsin-K-Deficient Mice. Proc. Natl. Acad. Sci. U.S.A. 95 (23), 13453–13458. 10.1073/pnas.95.23.13453 9811821 PMC24840

[B90] Sanchez-Fernandez M. A. Sbacchi S. Correa-Tapia M. Naumann R. Klemm J. Chambon P. (2012). Transgenic Mice for a Tamoxifen-Induced, Conditional Expression of the Cre Recombinase in Osteoclasts. PLoS One 7 (5), e37592. 10.1371/journal.pone.0037592 22624050 PMC3356310

[B91] Sauer B. (1998). Inducible Gene Targeting in Mice Using the Cre/loxSystem. Methods 14 (4), 381–392. 10.1006/meth.1998.0593 9608509

[B92] Shaw R. J. (2009). LKB1 and AMP-Activated Protein Kinase Control of mTOR Signalling and Growth. Acta Physiol. (Oxf). 196 (1), 65–80. 10.1111/j.1748-1716.2009.01972.x 19245654 PMC2760308

[B93] Shi G.-P. Sukhova G. K. Grubb A. Ducharme A. Rhode L. H. Lee R. T. (1999). Cystatin C Deficiency in Human Atherosclerosis and Aortic Aneurysms. J. Clin. Invest. 104 (9), 1191–1197. 10.1172/jci7709 10545518 PMC409823

[B94] Soki F. N. Yoshida R. Paglia D. N. Duong L. T. Hansen M. F. Drissi H. (2018). Articular Cartilage Protection in Ctsk ‐/‐ Mice Is Associated with Cellular and Molecular Changes in Subchondral Bone and Cartilage Matrix. J. Cell Physiol. 233 (11), 8666–8676. 10.1002/jcp.26745 29781506

[B95] Soukas A. Cohen P. Socci N. D. Friedman J. M. (2000). Leptin-specific Patterns of Gene Expression in White Adipose Tissue. Genes Dev. 14 (8), 963–980. 10.1101/gad.14.8.963 10783168 PMC316534

[B96] Sukhova G. K. Shi G. P. Simon D. I. Chapman H. A. Libby P. (1998). Expression of the Elastolytic Cathepsins S and K in Human Atheroma and Regulation of Their Production in Smooth Muscle Cells. J. Clin. Invest. 102 (3), 576–583. 10.1172/jci181 9691094 PMC508918

[B97] Suzuki M. Takahashi N. Sobue Y. Ohashi Y. Kishimoto K. Hattori K. (2020). Hyaluronan Suppresses Enhanced Cathepsin K Expression via Activation of NF-Κb with Mechanical Stress Loading in a Human Chondrocytic HCS-2/8 Cells. Sci. Rep. 10 (1), 216. 10.1038/s41598-019-57073-8 31937805 PMC6959248

[B98] Svelander L. Erlandsson-Harris H. Astner L. Grabowska U. Klareskog L. Lindstrom E. (2009). Inhibition of Cathepsin K Reduces Bone Erosion, Cartilage Degradation and Inflammation Evoked by Collagen-Induced Arthritis in Mice. Eur. J. Pharmacol. 613 (1-3), 155–162. 10.1016/j.ejphar.2009.03.074 19358841

[B99] Takito J. Inoue S. Nakamura M. (2018). The Sealing Zone in Osteoclasts: A Self-Organized Structure on the Bone. Int. J. Mol. Sci. 19 (4), 984. 10.3390/ijms19040984 PMC5979552 29587415

[B100] Tanaka M. Yamada H. Nishikawa S. Mori H. Ochi Y. Horai N. (2016). Joint Degradation in a Monkey Model of Collagen-Induced Arthritis: Role of Cathepsin K Based on Biochemical Markers and Histological Evaluation. Int. J. Rheumatol. 2016, 8938916. 10.1155/2016/8938916 26949397 PMC4754492

[B101] Tanaka M. Hashimoto Y. Hasegawa C. Deacon S. Eastell R. (2017). Antiresorptive Effect of a Cathepsin K Inhibitor ONO-5334 and its Relationship to BMD Increase in a Phase II Trial for Postmenopausal Osteoporosis. BMC Musculoskelet. Disord. 18 (1), 267. 10.1186/s12891-017-1625-y 28629344 PMC5477094

[B102] To T. T. Witten P. E. Huysseune A. Winkler C. (2015). An Adult Osteopetrosis Model in Medaka Reveals the Importance of Osteoclast Function for Bone Remodeling in Teleost Fish. Comp. Biochem. Physiology Part C Toxicol. Pharmacol. 178, 68–75. 10.1016/j.cbpc.2015.08.007 26334373

[B103] To T. T. Witten P. E. Renn J. Bhattacharya D. Huysseune A. Winkler C. (2012). Rankl-induced Osteoclastogenesis Leads to Loss of Mineralization in a Medaka Osteoporosis Model. Development 139 (1), 141–150. 10.1242/dev.071035 22096076

[B104] Usami Y. Gunawardena A. T. Francois N. B. Otsuru S. Takano H. Hirose K. (2019). Possible Contribution of Wnt‐Responsive Chondroprogenitors to the Postnatal Murine Growth Plate. J. Bone Mineral Res. 34 (5), 964–974. 10.1002/jbmr.3658 PMC6536347 30602070

[B105] Varjosalo M. Taipale J. (2008). Hedgehog: Functions and Mechanisms. Genes Dev. 22 (18), 2454–2472. 10.1101/gad.1693608 18794343

[B106] Walia B. Lingenheld E. Duong L. Sanjay A. Drissi H. (2018). A Novel Role for Cathepsin K in Periosteal Osteoclast Precursors during Fracture Repair. Ann. N.Y. Acad. Sci. 1415 (1), 57–68. 10.1111/nyas.13629 29479711

[B107] Walkley C. R. Qudsi R. Sankaran V. G. Perry J. A. Gostissa M. Roth S. I. (2008). Conditional Mouse Osteosarcoma, Dependent on P53 Loss and Potentiated by Loss of Rb, Mimics the Human Disease. Genes Dev. 22 (12), 1662–1676. 10.1101/gad.1656808 18559481 PMC2428063

[B108] Wang C. Inzana J. A. Mirando A. J. Ren Y. Liu Z. Shen J. (2016). NOTCH Signaling in Skeletal Progenitors Is Critical for Fracture Repair. J. Clin. Invest. 126 (4), 1471–1481. 10.1172/jci80672 26950423 PMC4811137

[B109] Wei W. Ren J. Yin W. Ding H. Lu Q. Tan L. (2020). Inhibition of Ctsk Modulates Periodontitis with Arthritis via Downregulation of TLR9 and Autophagy. Cell Prolif. 53 (1), e12722. 10.1111/cpr.12722 31737959 PMC6985664

[B110] Worthley D. L. Churchill M. Compton J. T. Tailor Y. Rao M. Si Y. (2015). Gremlin 1 Identifies a Skeletal Stem Cell with Bone, Cartilage, and Reticular Stromal Potential. Cell 160 (1-2), 269–284. 10.1016/j.cell.2014.11.042 25594183 PMC4436082

[B111] Xie H. Cui Z. Wang L. Xia Z. Hu Y. Xian L. (2014). PDGF-BB Secreted by Preosteoclasts Induces Angiogenesis during Coupling with Osteogenesis. Nat. Med. 20 (11), 1270–1278. 10.1038/nm.3668 25282358 PMC4224644

[B112] Xu R. Hu J. Zhou X. Yang Y. (2018). Heterotopic Ossification: Mechanistic Insights and Clinical Challenges. Bone 109, 134–142. 10.1016/j.bone.2017.08.025 28855144

[B113] Yamada H. Mori H. Nakanishi Y. Nishikawa S. Hashimoto Y. Ochi Y. (2019). Effects of the Cathepsin K Inhibitor ONO-5334 and Concomitant Use of ONO-5334 with Methotrexate on Collagen-Induced Arthritis in Cynomolgus Monkeys. Int. J. Rheumatol. 2019, 5710340. 10.1155/2019/5710340 30906325 PMC6397998

[B114] Yamashita T. Hagino H. Hayashi I. Hayashibara M. Tanida A. Nagira K. (2018). Effect of a Cathepsin K Inhibitor on Arthritis and Bone Mineral Density in Ovariectomized Rats with Collagen-Induced Arthritis. Bone Rep. 9, 1–10. 10.1016/j.bonr.2018.05.006 29992179 PMC6034140

[B115] Yang P. Lv S. Wang Y. Peng Y. Ye Z. Xia Z. (2018). Preservation of Type H Vessels and Osteoblasts by Enhanced Preosteoclast Platelet-Derived Growth Factor Type BB Attenuates Glucocorticoid-Induced Osteoporosis in Growing Mice. Bone 114, 1–13. 10.1016/j.bone.2018.05.025 29800693 PMC6309783

[B116] Yang W. Wang J. Moore D. C. Liang H. Dooner M. Wu Q. (2013). Ptpn11 Deletion in a Novel Progenitor Causes Metachondromatosis by Inducing Hedgehog Signalling. Nature 499 (7459), 491–495. 10.1038/nature12396 23863940 PMC4148013

[B117] Yang Y. Chen Q. Zhou S. Gong X. Xu H. Hong Y. (2020). Skeletal Phenotype Analysis of a Conditional Stat3 Deletion Mouse Model. J. Vis. Exp. 2020 (161), e61390. 10.3791/61390 32716374

[B118] Yang Y. Chung M. R. Zhou S. Gong X. Xu H. Hong Y. (2019). STAT3 Controls Osteoclast Differentiation and Bone Homeostasis by Regulating NFATc1 Transcription. J. Biol. Chem. 294 (42), 15395–15407. 10.1074/jbc.ra119.010139 31462535 PMC6802509

[B119] Zhou B. O. Yue R. Murphy M. M. Peyer J. G. Morrison S. J. (2014). Leptin-receptor-expressing Mesenchymal Stromal Cells Represent the Main Source of Bone Formed by Adult Bone Marrow. Cell Stem Cell 15 (2), 154–168. 10.1016/j.stem.2014.06.008 24953181 PMC4127103

[B120] Zhou H. Newnum A. B. Martin J. R. Li P. Nelson M. T. Moh A. (2011). Osteoblast/osteocyte-specific Inactivation of Stat3 Decreases Load-Driven Bone Formation and Accumulates Reactive Oxygen Species. Bone 49 (3), 404–411. 10.1016/j.bone.2011.04.020 21555004

